# Impact of symptoms by gender and age in Japanese subjects with irritable bowel syndrome with constipation (IBS-C): a large population-based internet survey

**DOI:** 10.1186/s13030-018-0131-2

**Published:** 2018-09-04

**Authors:** Masanori Kosako, Hiraku Akiho, Hiroto Miwa, Motoyori Kanazawa, Shin Fukudo

**Affiliations:** 1 Japan-Asia Clinical Development 1, Development, Astellas Pharma Inc., 2-5-1, Nihonbashi-Honcho, Chuo-ku, Tokyo, 103-8411 Japan; 2 Former employee of Astellas Pharma Inc., Tokyo, Japan; 3 Division of Gastroenterology, Department of Internal Medicine, Hyogo College of Medicine, Nishinomiya, Japan; 4 Department of Behavioral Medicine, Tohoku University Graduate School of Medicine, Sendai, Japan

**Keywords:** Irritable bowel syndrome (IBS), Constipation, Gender, Age, Abdominal bloating, Stress, Food, Epidemiology

## Abstract

**Background:**

Irritable bowel syndrome with constipation (IBS-C) is a representative psychosomatic disorder. Several pathophysiological factors have been linked to IBS symptoms such as the modulation of gastrointestinal motility, visceral hypersensitivity, dysregulation of the gut-brain axis, genetic and environmental factors, sequelae of infection, and psychosocial disorders. It is likely that biopsychosocial aspects of IBS-C underlie its gender and age effects. However, the influence of each symptom of IBS-C by gender and age is not well understood. We hypothesized that the expression rate of each IBS-C symptom in females and in subjects aged 20–49 years was higher than that of subjects who were male and aged 50–79 years.

**Methods:**

We conducted an internet survey of 30,000 adults from the general Japanese population. IBS-C subjects were asked to answer a questionnaire on the degree of anxiety, thoughts about bowel habits, and their dominant gastrointestinal symptoms together with exacerbation factors. The correlation between gender and age and IBS-C symptoms was analyzed.

**Results:**

When analyzed by gender, the expression rate of abdominal discomfort, abdominal distention, and abdominal fullness was significantly higher in female than male IBS-C subjects (66.5% vs. 58.7%, *p* < 0.05; 54.7% vs. 43.6%, *p* < 0.01; 18.9% vs. 9.6%, *p* < 0.01, respectively). When analyzed by age, the expression rate of abdominal distention and abdominal pain was significantly higher among IBS-C subjects aged 20–49 years than those aged 50–79 years (55.7% vs. 46.8%, *p* < 0.05; 36.6% vs. 20.6%, *p* < 0.001, respectively). In contrast, there was no gender or age differences with regard to the most common and bothersome symptom (abdominal bloating) among IBS-C subjects.

**Conclusions:**

The expression rate of some IBS-C symptoms was higher among females and those aged 20–49 years than males and those aged 50–79 years, respectively. It is important to understand the impact of symptoms by gender and age to evaluate the pathology of IBS-C from a biopsychosocial perspective.

**Trial registration:**

Although this survey was an anonymous internet survey, we obtained informed consent for the study as an online response. The disclosure of this study was approved by the Ethics Committee of Tohoku University Graduate School of Medicine (approval number: 2015–1-405).

## Background

Irritable bowel syndrome (IBS) is a functional disorder that is characterized by persistent or recurrent gastrointestinal (GI) symptoms. IBS mainly causes abdominal pain or discomfort and bowel movement disturbance, but is not associated with an organic disease that can explain these symptoms [1]. According to the Rome III diagnostic criteria [1], which are international criteria for functional GI disorders, IBS is defined as “symptom onset at least 6 months prior to diagnosis, and recurrent abdominal pain or discomfort for ≥ 3 days/month in the last 3 months. IBS is one kind of functional bowel disorder, associated with ≥ 2 of the 3 items described in brackets [1. Improvement with defecation, 2. Onset associated with a change in frequency of stool, 3. Onset associated with a change in form (appearance) of stool]”. While IBS is not a fatal disease, the symptoms associated with IBS restrict patients’ daily and social activities, and have been reported to significantly reduce patients’ quality of life (QOL) [2, 3]. Based on the stool pattern at a particular point, IBS is classified into 4 subtypes: constipation (IBS-C), diarrhea (IBS-D), mixed (IBS-M), or unsubtyped IBS (IBS-U) [1]. Among these subtypes, IBS-C is defined by hard or lumpy stools in ≥25% of bowel movements and loose (mushy) or watery stools in < 25% [1].

Several factors are thought to be related to IBS symptoms, including modulation of gastrointestinal motility, visceral hypersensitivity, dysregulated gut-brain axis, genetic and environmental factors, sequelae of infection, and psychosocial disorders. It is important to understand the disease from a biopsychosocial perspective.

Some reports have shown that the prevalence of IBS differs according to gender and age. Kanazawa et al. reported that approximately 16% of women and approximately 13% of men were diagnosed with IBS among survey subjects who received health check-ups [4]. A survey of the general Japanese population also found that the prevalence of IBS was significantly higher in women (1.7 times higher) than in men [5], which is consistent with data from other countries showing that the prevalence of IBS is generally higher in women. The prevalence of IBS is higher in individuals aged under 50 years than in those over 50 years [6]. IBS-C patients are often young and the percentage of female IBS-C patients is significantly higher than that of male IBS-C patients [7]. The higher prevalence of IBS in women compared with men may be associated with sex hormone fluctuations, which reportedly affect IBS symptoms, with symptoms appearing stronger before menstruation. Rectal susceptibility is also increased in women [8, 9]. Generally, visceral perception decreases with age, and Wilms et al. reported an age-related decrease in abdominal pain perception [10]. Psychological and somatic symptoms in IBS may be associated with sex hormones and menstruation, as well as sex-related genes and social gender differences [11, 12], the gender-specific psychological way of illness perception, symptom awareness and coping with the disease. However, the prevalence of each symptom of IBS-C by gender and age is not well understood. We previously conducted survey of the current status of IBS and found that the prevalence of IBS-C according to the Rome III diagnostic criteria was 2.8% and that abdominal bloating was the most bothersome symptom [13]. However, we did not investigate the impact of gender or age on IBS-C symptoms. Given that Internet use decreases with increasing age, data obtained from a large number of elderly IBS-C subjects such as in this survey will be valuable.

Based on previous reports, we hypothesized that the expression rate of each IBS-C symptom was higher in females and those aged 20–49 years than in males and those aged 50–79 years, respectively. We verified this hypothesis by analyzing an internet survey to investigate the prevalence of IBS-C symptoms by gender and age.

## Methods

This study used the same database as that published in our previous study [13] but conducted different hypotheses and analyses. While the aim of the previous study was to identify the most bothersome symptom in patients with IBS-C [13], the aim of the present study was to investigate the prevalence of IBS-C symptoms by gender and age. Details of the survey are described in the previous report [13]. Briefly, as a preliminary survey, 30,000 adults from the general Japanese population were recruited in October 28–31, 2013, for an internet survey to identify subtypes of IBS using a Macromill monitor panel (Macromill, Inc., Japan). Participants were asked to provide answers to questions regarding their living area, marriage status, children, income, and profession. Among the 30,000 participants, the screened subjects diagnosed with IBS-C using the Rome III criteria and the same number of age- and sex-matched non-IBS subjects randomly selected as controls were invited to participate in the main survey in November 1–4, 2013. In the main survey, IBS-C subjects were asked to answer a questionnaire on the degree of anxiety they experienced in their daily lives, the number of bowel movements they had in a week and thoughts about their bowel habits, and their dominant GI symptoms (including the most bothersome symptom) together with exacerbation factors, such as the circumstances and timing of symptoms and exacerbation. Degree of anxiety was assessed on a 4-point ordinate scale (0, Almost; 1, Often; 2, Sometimes; 3, None) based on the Rome III diagnostic questionnaire. The details of the questionnaires were provided in our previous report as additional files [13].

The correlation between gender and age and IBS-C symptoms was secondarily analyzed after publication of our previous report [13]. Given that IBS symptoms contains can vary, such as from GI symptoms to anxiety, we investigated differences in IBS symptoms between gender and age groups. Age was stratified into two groups, < 50 years and ≥ 50 years. Age ≥ 50 years was selected as the threshold because it was close to the median age of the IBS-C subjects in this survey and is considered a risk factor by the Japanese guidelines for treatment of IBS [12]. Comparisons between two groups were conducted using the Mann-Whitney U-test or χ^2^ test. Associations between the symptoms and exacerbation factors among IBS-C subjects were evaluated using Kendall’s τ-b. Analysis of multiplicity was not performed in the severity of the most bothersome symptom between two groups because each test was exploratory. Since the sample size of the age- and sex- matched subjects was low, analysis of age- and sex- matched subjects was not performed. The level of statistical significance was set at a *p*-value of less than 0.05.

## Results

### Gender

Five hundred and forty-one female and 218 male IBS-C subjects completed the consecutive questionnaires in the main survey. The demographics of participants by gender are shown in Table 1. The mean age ± standard deviation (SD) of female IBS-C subjects was significantly lower than that of males (47 ± 14 vs. 49 ± 15, *p* < 0.05). Female IBS-C subjects experienced lower frequency bowel movements (*p* < 0.001) and a lower ideal frequency of bowel movements (*p* < 0.05) than male IBS-C subjects. In contrast, there were no significant differences between female and male IBS-C subjects in whether participants considered bowel habit to be an indicator of health and in the degree of anxiety felt in daily life (Table 1). Professions of IBS-C subjects by gender are shown in Table 2.

**Table 1:** Characteristics of subjects with irritable bowel syndrome with constipation and their beliefs regarding bowel habits

	Female (*n* = 541)	Male (*n* = 218)	*p*-value	Age 20–49 years (*n* = 404)	Age 50–79 years (*n* = 355)	*p*-value
Female/male (n)	541/0	0/218	N/A	298/106 (73.8/26.2)	243/112 (68.5/31.5)	n.s.
Age (mean ± SD, years)	47 ± 14	49 ± 15	< 0.05	36 ± 8	60 ± 7	N/A
Frequency of bowel movements (median, times/week)	3	5	< 0.001	3	4	< 0.05
Ideal frequency of bowel movements			< 0.05			n.s.
6 times/week or less	123 (22.7)	42 (19.3)		91 (22.5)	74 (20.8)	
7 times/week	402 (74.3)	161 (73.9)		299 (74.0)	264 (74.4)	
8 times/week or more	16 (3.0)	15 (6.9)		14 (3.5)	17 (4.8)	
Consider bowel habits to be an indicator of health			n.s.			n.s.
None	50 (9.2)	21 (9.6)		29 (7.2)	42 (11.8)	
Sometimes	160 (29.6)	61 (28.0)		114 (28.2)	107 (30.1)	
Often	124 (22.9)	62 (28.4)		100 (24.8)	86 (24.2)	
Mostly	104 (19.2)	44 (20.2)		80 (19.8)	68 (19.2)	
Always	103 (19.0)	30 (13.8)		81 (20.0)	52 (14.6)	
Degree of anxiety in daily life			n.s.			< 0.001
None	126 (23.3)	50 (22.9)		70 (17.3)	106 (29.9)	
Sometimes	309 (57.1)	131 (60.1)		225 (55.7)	215 (60.6)	
Often	80 (14.8)	31 (14.2)		84 (20.8)	27 (7.6)	
Almost	26 (4.8)	6 (2.8)		25 (6.2)	7 (2.0)	

Data are expressed as n (%) unless otherwise indicated

**Table 2:** Profession of IBS-C subjects

Profession	Female (n = 541)	Male (n = 218)	Age 20–49 years (n = 404)	Age 50–79 years (n = 355)
Civil servant	7 (1.3)	9 (4.1)	7 (1.7)	9 (2.5)
Manager and officer	1 (0.2)	6 (2.8)	2 (0.5)	5 (1.4)
Employee (administrative office)	69 (12.8)	37 (17.0)	73 (18.1)	33 (9.3)
Employee (engineering)	6 (1.1)	31 (14.2)	25 (6.2)	12 (3.4)
Employee (other)	20 (3.7)	23 (10.6)	30 (7.4)	13 (3.7)
Self-employed	20 (3.7)	20 (9.2)	17 (4.2)	23 (6.5)
Freelance	5 (0.9)	6 (2.8)	2 (0.5)	9 (2.5)
Homemaker	229 (42.3)	2 (0.9)	106 (26.2)	125 (35.2)
Temporary work	103 (19.0)	21 (9.6)	74 (18.3)	50 (14.1)
Student	12 (2.2)	10 (4.6)	22 (5.4)	0 (0.0)
Other	27 (5.0)	19 (8.7)	19 (4.7)	27 (7.6)
Not employed	42 (7.8)	34 (15.6)	27 (6.7)	49 (13.8)

Data are expressed as n (%) unless otherwise indicated

The degree of anxiety was significantly associated with abdominal discomfort (τ = 0.11, *p* < 0.05) and abdominal pain (τ = 0.13, *p* < 0.05) but not abdominal bloating (Kendall’s τ = 0.08, n.s.) in female IBS-C subjects. In contrast, the degree of anxiety was not significantly associated with abdominal bloating (τ = 0.09, n.s.) abdominal discomfort (τ = 0.04, n.s.), or abdominal pain (τ = 0.06, n.s.) in male IBS-C subjects (Table 3).

**Table 3:** Association between the degree of anxiety and GI symptoms in IBS-C subjects

GI symptoms/Degree of anxiety	Female					Male				
	None	Sometimes	Often	Almost	Kendall’s τ	None	Sometimes	Often	Almost	Kendall’s τ
Abdominal bloating					0.08					0.09
No	30	62	9	5		12	29	4	0	
Yes	96	247	71	21		38	102	27	6	
Abdominal discomfort					0.11*					0.04
No	55	97	22	7		21	56	12	1	
Yes	71	212	58	19		29	75	19	5	
Abdominal pain					0.13*					0.06
No	95	223	47	12		37	100	23	1	
Yes	31	86	33	14		13	31	8	5	
	Age 20–49 years					Age 50–79 years				
Abdominal bloating					0.12*					0.05
No	19	52	9	4		23	39	4	1	
Yes	51	173	75	21		83	176	23	6	
Abdominal discomfort					0.09					0.08
No	28	79	22	7		48	74	12	1	
Yes	42	146	62	18		58	141	15	6	
Abdominal pain					0.13*					−0.01
No	50	148	49	9		82	175	21	4	
Yes	20	77	35	16		24	40	6	3	

*GI* gastrointestinal

*p < 0.05, the Kendall’s τ-b

**Fig. 1:**
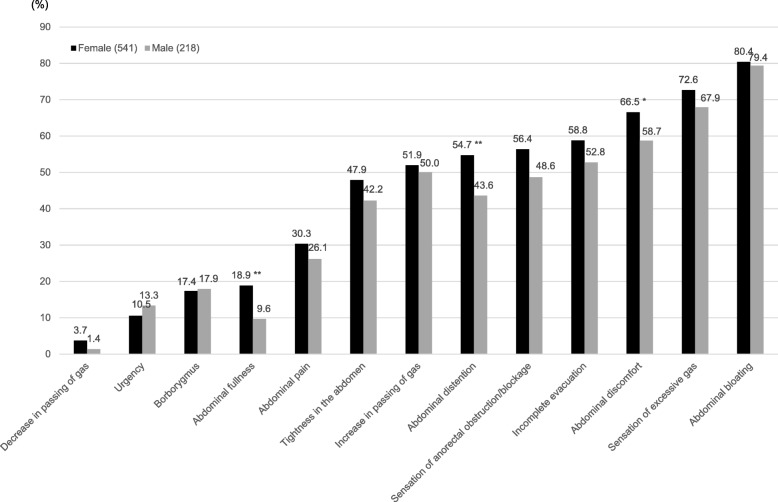
Rate of gastrointestinal symptoms in female and male IBS-C subjects. *p < 0.05, **p < 0.01 vs males

The expression rate of GI symptoms in female and male IBS-C subjects is shown in Fig. 1. Although abdominal bloating was the most common symptom associated with constipation in IBS-C subjects of both genders (female 80.4%, male 79.4%), the expression rate of almost all IBS symptoms was slightly higher among females than males. In particular, the expression rate of abdominal discomfort, abdominal distention, and abdominal fullness was significantly higher among female than male IBS-C subjects (66.5% vs. 58.7%, *p* < 0.05; 54.7% vs. 43.6%, *p* < 0.01; 18.9% vs. 9.6%, *p* < 0.01, respectively) (Fig. 1).

**Fig. 2:**
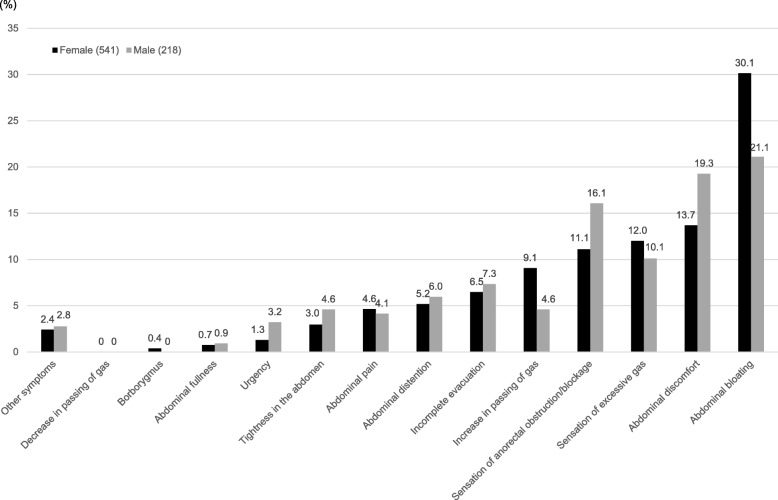
Rate of the most bothersome symptom in female and male IBS-C subjects

The expression rate of the most bothersome symptom in female and male IBS-C subjects is shown in Fig. 2. Abdominal bloating was reported as the most bothersome symptom in both female and male IBS-C subjects (female 30.1%, male 21.1%) (Fig. 2), and was most likely to occur after a meal (female 54.6%, male 43.5%) (Table 4). The most bothersome symptom (abdominal pain) in female and male subjects was most likely to occur during menstruation (48.0%) and at work/school (44.4%), respectively (Table 4). There was no significant difference in the severity of the most bothersome symptom (abdominal bloating and abdominal pain) between female and male IBS-C subjects (Table 5). In contrast, the severity of the most bothersome symptom (abdominal discomfort) was significantly greater in female than male IBS-C subjects (*p* < 0.05).

**Table 4:** Occurrence of the “most bothersome symptoms” in IBS-C subjects

Situation/Symptom	Abdominal bloating				Abdominal discomfort				Abdominal pain			
	Female (*n* = 163)	Male (*n* = 46)	Age 20–49 years (*n* = 104)	Age 50–79 years (*n* = 105)	Female (*n* = 74)	Male (*n* = 42)	Age 20–49 years (*n* = 66)	Age 50–79 years (*n* = 50)	Female (*n* = 25)	Male (*n* = 9)	Age 20–49 years (*n* = 24)	Age 50–79 years (*n* = 10)
On the way to work/school by bus or train	16.6	23.9	25.0	11.4	17.6	28.6	30.3	10.0	8.0	0	8.3	0
At work/school	26.4	39.1	36.5	21.9	23.0	31.0	34.8	14.0	24.0	44.4	29.2	30.0
During a conference presentation/exam	3.7	13.0	5.8	5.7	9.5	11.9	15.2	4.0	12.0	0	12.5	0
After drinking alcohol	1.2	6.5	3.8	1.0	8.1	4.8	9.1	4.0	0	0	0	0
After drinking milk	2.5	4.3	1.9	3.8	6.8	2.4	4.5	6.0	12.0	11.1	16.7	0
After a meal	54.6	43.5	51.9	52.4	50.0	31.0	40.9	46.0	32.0	11.1	20.8	40.0
On a sightseeing trip	28.2	10.9	18.3	30.5	16.2	9.5	9.1	20.0	12.0	0	8.3	10.0
On a business trip	2.5	6.5	3.8	2.9	5.4	9.5	7.6	6.0	4.0	0	4.2	0
During times of stress	26.4	28.3	31.7	21.9	33.8	28.6	36.4	26.0	28.0	33.3	33.3	20.0
After taking medication	3.1	2.2	1.9	3.8	1.4	2.4	3.0	0	4.0	11.1	8.3	0
During menstruation (females only)	11.7	0	14.4	3.8	16.2	0	18.2	0	48.0	0	45.8	10.0

Data are expressed as rate (%)

**Table 5:** Severity of the “most bothersome symptoms” in IBS-C subjects

GI symptom	0: Very mild	1: Mild	2: Moderate	3: Severe	4: Very Severe	Median	*p*-value
Abdominal bloating							n.s
Female (n = 163)	0.6	4.3	31.9	46.6	16.6	3	
Male (n = 46)	2.2	15.2	32.6	39.1	10.9	2.5	
Abdominal discomfort							< 0.05
Female (n = 74)	1.4	2.7	40.5	36.5	18.9	3	
Male (n = 42)	0	16.7	54.8	19.0	9.5	2	
Abdominal pain							n.s.
Female (n = 25)	4.0	8.0	36.0	40.0	12.0	3	
Male (n = 9)	0	11.1	22.2	55.6	11.1	3	
Abdominal bloating							n.s
Age 20–49 years (n = 104)	1.0	7.7	33.7	40.4	17.3	3	
Age 50–79 years (n = 105)	1.0	5.7	30.5	49.5	13.3	3	
Abdominal discomfort							< 0.05
Age 20–49 years (n = 66)	0	1.5	48.5	28.8	21.2	2.5	
Age 50–79 years (n = 50)	2.0	16.0	42.0	32.0	8.0	2	
Abdominal pain							n.s.
Age 20–49 years (n = 24)	4.2	4.2	37.5	41.7	12.5	3	
Age 50–79 years (n = 10)	0	20.0	20.0	50.0	10.0	3	

*GI* gastrointestinal

Data are expressed as rate (%)

### Age

Four hundred and four IBS-C subjects aged 20–49 years and 355 aged 50–79 years completed the consecutive questionnaires. The demographics of the participants in each age group are shown in Table 1. The mean age ± SD of participants in the two age groups was 36 ± 8 and 60 ± 7 years. IBS-C subjects aged 20–49 years experienced lower frequency bowel movements (*p* < 0.05) and a higher degree of anxiety in their daily lives (*p* < 0.001) than those aged 50–79 years. In contrast, there was no significant difference between the two age groups in the ideal frequency of bowel movements, or participants’ consideration of whether bowel habit was an indicator of health (Table 1). Professions of IBS-C subjects by age are shown in Table 2.

The degree of anxiety was significantly associated with abdominal bloating (Kendall’s τ = 0.12, *p* < 0.05) and abdominal pain (τ = 0.13, *p* < 0.05) but not abdominal discomfort (τ = 0.09, n.s.) in IBS-C subjects aged 20–49 years. In contrast, the degree of anxiety was not significantly associated with abdominal bloating (τ = 0.05, n.s.) abdominal discomfort (τ = 0.08, n.s.), or abdominal pain (τ = − 0.01, n.s.) in IBS-C subjects aged 50–79 years (Table 3).

**Fig. 3:**
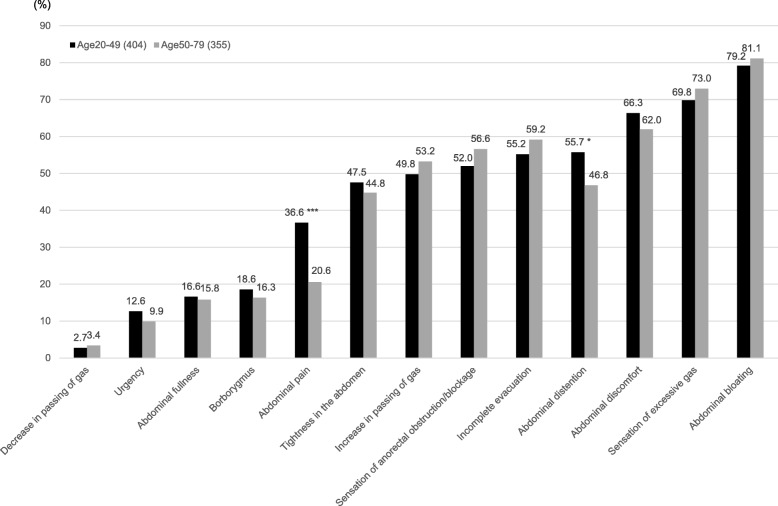
Rate of gastrointestinal symptoms in IBS-C subjects aged 20–49 and 50–79 years. *p < 0.05, **p < 0.01, ***p < 0.001 vs age 50–79 years

The expression rate of GI symptoms in IBS-C subjects aged 20–49 and 50–79 years is shown in Fig. 3. Although the most common symptom associated with constipation was abdominal bloating in both age groups (age 20–49 years 79.2%, age 50–79 years 81.1%), the expression rate of abdominal distention and abdominal pain was significantly higher among IBS-C subjects aged 20–49 years than those aged 50–79 years (55.7% vs. 46.8%, *p* < 0.05; 36.6% vs. 20.6%, *p* < 0.001, respectively) (Fig. 3).

**Fig. 4:**
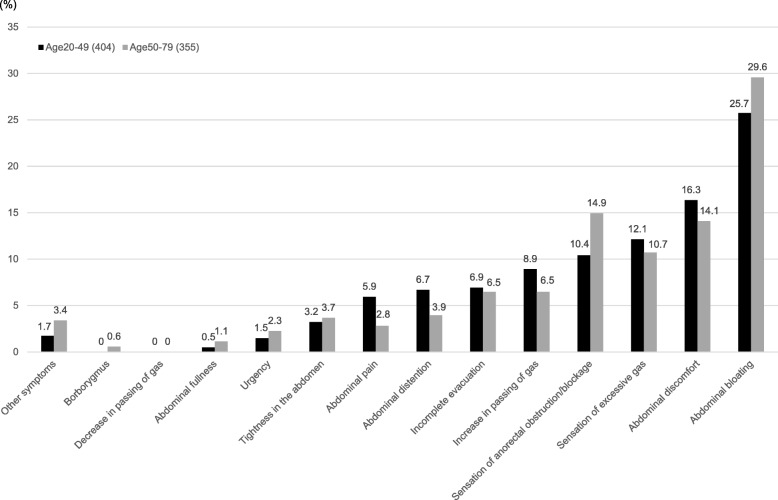
Rate of the most bothersome symptom in IBS-C subjects aged 20–49 and 50–79 years

The expression rate of the most bothersome symptom among IBS-C subjects aged 20–49 and 50–79 years is shown in Fig. 4. The most bothersome symptom among both age groups was abdominal bloating (age 20–49 years 25.7%, age 50–79 years 29.6%) (Fig. 4), and was most likely to occur after a meal (age 20–49 years 51.9%, age 50–79 years 52.4%) (Table 4). Occurrence of the most bothersome symptom (abdominal pain) in IBS-C subjects aged 20–49 and 50–79 years was most likely to occur during menstruation (45.8%) and after a meal (40.0%), respectively (Table 4). There was no significant difference in the severity of the most bothersome symptom (abdominal bloating and abdominal pain) between the two age groups (Table 5). In contrast, the severity of the most bothersome symptom (abdominal discomfort) was significantly greater among IBS-C subjects aged 20–49 years than those aged 50–79 years (*p* < 0.05).

## Discussion

To our knowledge, this is the first study to report the expression of IBS-C symptoms in the Rome III by gender and age. We observed that the expression rate of abdominal discomfort, abdominal distention, and abdominal fullness was significantly higher among female than male IBS-C subjects. Moreover, the expression rate of abdominal distention and abdominal pain was significantly higher among IBS-C subjects aged 20–49 than 50–79 years. A study by Adeyemo et al. on overall IBS reported that female IBS subjects showed a greater prevalence of constipation-associated symptoms, particularly bloating and abdominal distension, than male IBS patients [11]. Studies reporting differences in the prevalence of symptoms between female and male IBS-C patients suggest that increased visceral sensitivity and sex hormones may be associated with the expression rate of IBS-C symptoms [7, 14, 15]. IBS-C symptoms may be associated with the gender-specific psychological way of illness perception, symptom awareness and coping with the disease. Visceral perception of abdominal pain in IBS-C patients may also decrease with age.

Our results showed that the most common symptom and the most bothersome symptom (abdominal bloating for both) did not differ by gender or age. Because we assumed that the Japanese term for “bloating” consisted of “abdominal bloating”, “abdominal distention”, “sensation of excessive gas”, “tightness in the abdomen” and “abdominal fullness”, we also surveyed the frequency of occurrence of these 5 specific symptoms in IBS-C subjects. Abdominal bloating is associated with decreased QOL and may lead to a higher frequency of physician visits [16]. Abdominal bloating is considered a key symptom among IBS patients in Asia [17], and may be an important reason prompting IBS-C patient consultations in Japan.

We previously reported that the most bothersome symptom (abdominal bloating) among IBS-C subjects was most likely to occur after a meal. This result did not change when subjects were stratified by gender and age. IBS symptoms such as abdominal pain and bloating occur or are exacerbated postprandially in approximately two-thirds of patients [18, 19]. It is possible that the administration of IBS-C medications before meals will prevent the worsening of abdominal symptoms associated with anxiety. Young IBS-C subjects had a higher degree of anxiety in their daily lives than elderly subjects, while anxiety was similar between genders. In contrast, the degree of anxiety was correlated with some GI symptoms (abdominal bloating, abdominal discomfort, and abdominal pain) in young and female IBS-C subjects but not in elderly and male IBS-C subjects. Therefore, young IBS-C patients may be more suitable candidates for the biopsychosocial approach.

The most bothersome symptom (abdominal pain) in female IBS-C subjects and those aged 20–49 years occurred predominantly during menstruation. Houghton et al. reported that the menses in IBS is associated with a worsening of abdominal pain [9]. Together with our findings, this suggests that menses in female and young IBS-C subjects may be associated with a worsening of abdominal pain. In contrast, the most bothersome symptom (abdominal pain) in male IBS-C subjects occurred predominantly at work/school and during times of stress. Although more female IBS-C subjects were homemakers compared with male IBS-C patients, male IBS-C subjects may feel more stress than female IBS-C subjects in their daily life. The severity of the most bothersome symptom (abdominal discomfort) in female and 20–49-year-old IBS-C subjects was significantly greater than that in male and 50–79-year old IBS-C subjects, respectively. Chang et al. reported that a number of gender-related factors may impact the clinical symptoms and response of IBS, such as gender roles, sociocultural differences, hormonal effects such as menstrual cycle variation, and biological differences influencing gut function and treatment response [20]. Suprathreshold distention of the rectum using a barostat induces abdominal discomfort and correspondingly causes higher activation of the medial prefrontal cortex and pregenual anterior cingulate cortex in IBS patients than in healthy controls [21]. Specifically, increasing age is associated with functional alterations in the blood-oxygen-level-dependent signal in the anterior cingulate cortex and medial prefrontal cortex [22]. It is likely that biopsychosocial aspects of IBS-C underlie its gender and age effects.

Several limitations of our study warrant mention. First, our study used an internet survey rather than a mail survey with random sampling from a list of residential areas in Japan. Subjects who are interested in their health may be more likely to participate in an internet survey. However, we collected a relatively large (30,000 participants) sample from a large monitor panel throughout Japan. Further, ascertainment bias is unlikely because previous reports indicate that the prevalence of overall IBS and IBS-C is similar to that of overall IBS and IBS-C using the Rome III diagnostic criteria in Japan [7, 23] and other countries [24]. Second, we could not exclude organic GI diseases and/or other comorbid diseases because we did not collect data on objective examination, prescription GI and psychiatric drugs, or those for other disease. IBS subjects have more somatic/psychiatric comorbidities that can affect their daily lives than non-IBS subjects [25]. However, the occurrence of abdominal discomfort or abdominal pain in our study population was similar to that in a previously reported population that excluded subjects with physician-diagnosed lower GI disorders [26], suggesting that organic GI diseases and/or other comorbid diseases likely had little effect on our findings. Moreover, our results did not include subjects aged < 20 years, marital status, and education. The results for anxiety are limited by the use of a single item. Furthermore, the Rome III criteria were revised to Rome IV in May 2016, after this internet survey was conducted [27]. The main changes in the Rome IV criteria for IBS were the symptomatic frequency (Rome IV: ≥ 1 day per week, Rome III: ≥ a few days per month) and the presence of abdominal pain (Rome IV: abdominal pain, Rome III: abdominal pain or discomfort). Future research based on the Rome IV criteria is warranted.

## Conclusion

A large population-based internet survey suggests that the expression rate of some IBS-C symptoms is higher among female IBS-C subjects and those aged 20–49 years than among males and those aged 50–79 years, respectively. The degree of anxiety correlated with some GI symptoms (abdominal bloating, abdominal discomfort, and abdominal pain) in young and female IBS-C subjects but not in elderly and male IBS-C subjects. It is important to understand the impact of IBS-C symptoms by gender and age to evaluate the pathology of IBS-C from a biopsychosocial perspective.

## Abbreviations

IBS: Irritable bowel syndrome
IBS-C: IBS with constipation
IBS-D: IBS with diarrhea
IBS-M: Mixed IBS
IBS-U: Unspecified IBS
GI: Gastrointestinal
QOL: Quality of life
